# How Does Conversion of Natural Tropical Rainforest Ecosystems Affect Soil Bacterial and Fungal Communities in the Nile River Watershed of Uganda?

**DOI:** 10.1371/journal.pone.0104818

**Published:** 2014-08-12

**Authors:** Peter O. Alele, Douglas Sheil, Yann Surget-Groba, Shi Lingling, Charles H. Cannon

**Affiliations:** 1 Key Laboratory of Tropical Forest Ecology, Xishuangbanna Tropical Botanical Garden (XTBG), Chinese Academy of Sciences, Kunming, Yunnan, P. R. China; 2 University of the Chinese Academy of Sciences, Beijing, P. R. China; 3 Great Nile Conservation Centre (GNCC), Lira, Uganda; 4 Department of Ecology and Natural Resource Management, Norwegian University of Life Sciences, Ås, Norway; 5 Center for International Forestry Research (CIFOR), Bogor, Indonesia; 6 Department of Ecology and Natural Resource Management, School of Environment, Science and Engineering, Southern Cross University, Lismore, New South Wales, Australia; 7 Institute of Tropical Forest Conservation (ITFC), Mbarara University of Science and Technology (MUST), Kabale, Uganda; 8 Texas Tech University, Lubbock, Texas, United States of America; Bangor University, United Kingdom

## Abstract

Uganda's forests are globally important for their conservation values but are under pressure from increasing human population and consumption. In this study, we examine how conversion of natural forest affects soil bacterial and fungal communities. Comparisons in paired natural forest and human-converted sites among four locations indicated that natural forest soils consistently had higher pH, organic carbon, nitrogen, and calcium, although variation among sites was large. Despite these differences, no effect on the diversity of dominant taxa for either bacterial or fungal communities was detected, using polymerase chain reaction-denaturing gradient gel electrophoresis (PCR-DGGE). Composition of fungal communities did generally appear different in converted sites, but surprisingly, we did not observe a consistent pattern among sites. The spatial distribution of some taxa and community composition was associated with soil pH, organic carbon, phosphorus and sodium, suggesting that changes in soil communities were nuanced and require more robust metagenomic methods to understand the various components of the community. Given the close geographic proximity of the paired sampling sites, the similarity between natural and converted sites might be due to continued dispersal between treatments. Fungal communities showed greater environmental differentiation than bacterial communities, particularly according to soil pH. We detected biotic homogenization in converted ecosystems and substantial contribution of β-diversity to total diversity, indicating considerable geographic structure in soil biota in these forest communities. Overall, our results suggest that soil microbial communities are relatively resilient to forest conversion and despite a substantial and consistent change in the soil environment, the effects of conversion differed widely among sites. The substantial difference in soil chemistry, with generally lower nutrient quantity in converted sites, does bring into question, how long this resilience will last.

## Introduction

Tropical rainforests (TRF) possess most of the world's terrestrial biodiversity and deforestation is the leading cause of biodiversity loss [1], [2]. Due to their high biodiversity and endemism, the tropical rainforests in Uganda's Nile river watershed are among the world's most important for their conservation values. But these areas are under pressure. The United Nations Population Division [3] predicts that the population of the Nile Basin states will increase by 57% from 2010 to 2030, reaching 647 million people. This rapid population growth, high levels of poverty and prevalent civil insecurity continue to exert severe pressure on natural resources in the region. Uganda in particular has one of the world's highest population growth rates (3.2% per year) [4]. Most of this growing population (nearly 80%) is dependent on agriculture leading to large scale and continuing conversion of natural habitats [5].

Soil communities form the foundation of any ecosystem, in terms of nutrient cycling and availability, so understanding how land conversion affects these communities is an important first step. The effect of land use change on soil microbial communities has been studied in South American and Southeast Asian forests [6], [7], but not in the biodiversity hotspots of the Nile river watershed. There is considerable global concern about the loss of biodiversity and the consequences for human well-being [8]. Microorganisms in particular play a vital role in many ecological processes and environmental services [9]: these roles are not always apparent or well characterized but if all microbes died the world would rapidly become buried in undecomposed dead material. Due to their significance in maintaining ecosystem function and productivity [9], [10], our study offers a vital exploratory appraisal of microbial community dynamics in natural TRF and human-converted sites. We don't know if there are reasons to be concerned unless we look. Developing such knowledge is critical at this point, because populations in the Nile river watershed are highly dependent on forests for basic requirements such as food and fuel wood, with the environment contributing between 40–60% of the gross domestic product (GDP) of the Nile riparian states [11].

Because of widespread loss of biodiversity, focus from species conservation within particular habitats has been shifted to conservation of communities [12], [13]. It is therefore important to explore and understand how composition and diversity changes across spatial scales in a given context [14]–[16]. Changes in ecosystems caused by conversion to intensive management can lead to biotic homogenization, the increase in community similarity over time and/or space and an implied loss of rare and vulnerable taxa when examined at larger scales [17]–[19]. Because microorganisms are the most diverse organisms on earth with most taxa and respective functions and behaviors as yet unknown, determining their sensitivities and biogeography remains a major challenge. But in the longer term such knowledge will help us better understand the sustainability of land-use systems and associated environmental values.

This study was therefore necessary as a first step in exploring these relationships, and to enhance understanding so as to contribute to the informed and appropriate stewardship of the region's natural resources. Our objective was to establish how forest conversion and soil factors affect soil bacterial and fungal diversity and community composition in the tropical rain-forests in the Nile river watershed of Uganda. We chose four forest sites found within protected areas, with paired treatments within each forest; (1) natural and (2) converted ecosystem sites. The natural forest ecosystem at each site had suffered minimal human disturbance, while converted areas had been transformed to cropland. These matched sites found in different locations and environmental conditions each experienced different land use histories, conservation circumstances and individual challenges for management.

In each matched set of natural and converted sites, we compared soil physical and chemical properties and microbial community diversity and composition using standard PCR-based genotyping techniques. We then calculated community similarity indices between sites. This approach would allow us to examine both environmental and biotic changes in the soil community associated with conversion. Disturbances of sufficient magnitude or duration may alter an ecosystem and force a different regime of predominant processes and structures that favor some populations over others [20].

We tested the null hypotheses that there was no difference in soil properties, band-types, and diversity between treatments. The influence of soil properties on microbial community diversity was measured by discriminant analysis and canonical correspondence analysis (CCA), [21], [22]. Because additive partitioning of diversity provides a useful framework for quantifying the spatial patterns of diversity across hierarchical spatial scales [23], [24], we partitioned total diversity (γ) in each ecosystem type (natural and converted) into additive components representing within-community diversity (α) and between-community diversity (β). Our objective was to identify the most important sources of total diversity so as to propose conservation measures for microbial communities in the TRF ecosystems of the Nile river watershed of Uganda.

## Methods

### Site description

**Figure 1:**
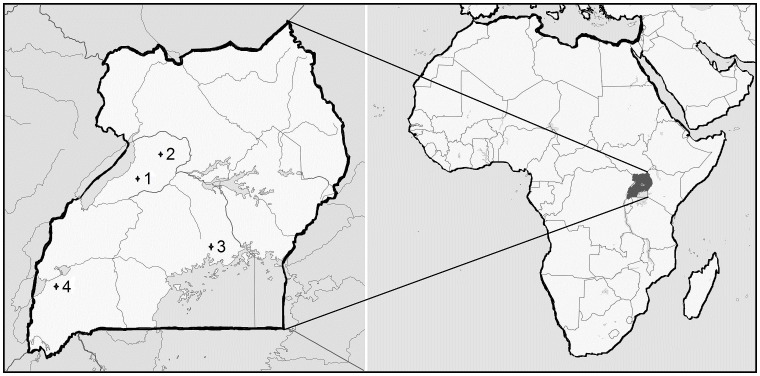
Map of Uganda showing the distribution of sampling sites; Budongo forest (1), Kaniyo Pabidi (2), Mabira forest (3), and Maramagambo forest (4).

We selected four tropical rainforest (TRF) sites because of their relative size, biodiversity, socio-economic and scientific importance (Fig 1). Mabira forest is located between the highly populated and urbanized Kampala city on the western side; the extensive and mechanized Lugazi sugar and tea plantations on the Eastern; and Lake Victoria on the southern side. Budongo forest is located next to the extensive Kinyala sugar plantations on one side and a densely populated mainly subsistence population scattered around it. Maramagambo and Kaniyo Pabidi are located within Queen Elizabeth and Murchison Falls national parks (NP) respectively. These two NP forests had perhaps the best protection due to presence of Uganda Wildlife Authority (UWA) personnel. However, Maramagambo's location starting on the steep slopes of the rift valley subjected it to frequent storms with strong runoff flow that swept away most of its top soil (Table 1).

**Table 1:** **Summary of study site description.**

Forest Site	Location	Size (km^2^)	Altitude (masl)	Geology	Forest type	Habitat type	Ecosystem description
Budongo	31°N 35° E 1°S 45° N	793	700–1,270	Weathered pre-cambium with ferrallitic sandy clay loams	Ironwood forest (*Cynometra alexandri*); Mixed forest (*Maesopis*), and colonizing forest (*Entandrophragma*)	Primary forest	Consists of a medium altitude moist semi-deciduous forest with areas of savanna and woodland. Converted areas consisted of deforested agricultural land being cultivated and planted with maize, beans, sweet potatoes and cassava. This land has existed as agricultural land for at least 15years.
Mabira	33° 0.00' E 0° 30.00' N	300	1,070–1,340	Ferrallitic soils with mainly sandy clay loams	Mixed forest	Secondary forest, heavily influenced by humans	The forest is surrounded by a densely populated area. Converted areas were actively cultivated and used to grow maize, groundnuts, beans, yams, cassava, sweet potatoes and a few scattered plants of coffee and sugarcane. The converted land had existed as agricultural land for at least 10 years.
Maramagambo	00° 33' 00" S and 29° 53' 00" E	1,978 (QENP)	910–1390	Ferrallitic soils with undifferentiated dark horizons	Medium altitude, moist, semi-deciduous forest	Secondary forest influenced by wildlife and humans	Forms part of the Queen Elizabeth NP (QENP) which is 1,978 km2. Converted areas consisted of cultivated and grazed areas with gardens of sweet potatoes, beans, maize, and sorghum with areas commonly grazed by cattle and goats.
Kaniyo Pabidi	Lat 1.916667 Long 31.666667	N/A	700–1,270	Freely drained ferruginous tropical soils	Mixed forest	Primary forest	Located north of Budongo forest and part of Murchison Falls N.P. Converted areas consisted of cultivated areas with crops like maize, beans, cassava and sweet potatoes.

[5], [61], [70], [71].

### Soil sampling design

We collected 400 core soil samples within 40 plots (1000 m^2^ each) in four TRF sites (Fig 1). We sampled five plots from each site of the natural TRF and five plots from the converted TRF. We established the plots at least 100 m from the ecosystem edge and 500 m apart and collected 10 evenly placed core subsamples of top soil (0–15 cm) from each plot and homogenized them into one sample per plot. We then derived a 500 g composite sample from the mixture, sieved and packed it for physical and chemical analyses and DNA extraction.

### Sample preparation

We sieved 100 g of the soil on-site through a 4 mm mesh, transported it to the laboratory on ice, and stored in a freezer at −40°C prior to nucleic acid extraction and analysis. We kept the rest of the soil for drying and physical and chemical analysis. We performed DNA extractions from 1 g of soil using the Ultra Clean soil DNA kit (Mo Bio Labs, Solana Beach, CA, USA) following the manufacturer's protocol. The purified DNA was detected by agarose gel electrophoresis, and the DNA was amplified by polymerase chain reaction (PCR).

### Soil property analyses

We measured the soil pH in 2.5∶1 water to soil suspension using a pH meter (10 g soil+ 25 ml of distilled water, shaken for 30 min and read on a calibrated pH meter). We then used the Walkley and Black method [25] to analyze soil organic carbon (SOC) and the Kjeldahl method [26] to determine soil nitrogen. We measured the soil phosphorus by the Bray and Kurtz no. 1 method [27]. The photoelectric flame photometer was used to determine the soil potassium, sodium and calcium after extraction with neutral ammonium acetate. We used the atomic absorption spectrometer to measure the soil magnesium after extraction with neutral ammonium acetate. The Bouyoucos hydrometer method adopted from Gee and Bauder [28] was used to determine soil texture. The soil copper and iron were then determined using the atomic absorption spectrometer after extraction with EDTA.

### PCR amplification and DGGE analysis

Polymerase chain reaction-denaturing gradient gel electrophoresis (PCR-DGGE) method has been used extensively in microbial ecology and is a robust and cost effective method for exploratory classification of microbial communities [29]. Following soil DNA extraction, we performed a PCR for each DNA extraction to amplify the 16S rRNA genes for bacteria and 18S rRNA genes for fungi using universal primers (Table 2).

**Table 2:** **Sequences of primers used in study.**

Microorganism	Primer	Sequence (5'–3')	Reference
Bacteria	F357	CGC CCG CCG CGC GCG GCG GGC GGG GCG GGG GCA CGG GGG GCC TAC GGG AGG CAG CAG	[72]
	907R	CCG TCA ATT CMT TTG AGT TT	
Fungi	FF390	CGA TAA CGA ACG AGA CCT	[73]
	FR1GC	AIC CAT TCA ATC GGT AIT	

PCR reactions had a final volume of 25 µl containing a final concentration of 1× TaKaRa ExTaq PCR buffer with MgCl_2_, 300 pM of primers for bacteria. We then added 200 µM dNTPs, 2.5 U ExTaq DNA polymerase (TaKaRa Bio, Otsu, Japan) and milliQ H_2_O to complete the volume, BSA was also added for the fungal community analysis. We performed PCR cycles with an initial denaturing temperature of 95 °C for 5 min, followed by 35 cycles of 95 °C for 30 sec, annealing temperature of 50 °C for 30 sec, extension of 72 °C for 1 min; and a final extension of 72 °C for 10 min. We checked the product of the PCR-rounds and quantified by agarose gel-electrophoresis.

We then performed 16S rRNA and 18S rRNA-DGGE analysis using a universal mutation detection system (Dcode Bio-Rad, Richmond, CA, USA) with a 6% and 8% acrylamide gel for bacteria and fungi respectively containing a gradient of 40–60% denaturant (100% denaturant contains 7 mol urea and 40% formamide). We applied 100 ng of PCR samples to the DGGE gel. DGGE was performed in 1 × TAE Buffer (40 mol Tris/acetate, pH 8; 1 mol ethylene diaminetetra acetic acid) at 60 °C and a constant voltage of 60 V for 16 hours. After staining with SYBR Green1, we recorded the DGGE gels as digital images and analyzed the DNA band numbers using image-processing software after subtracting background noise.

### Data analysis

We used the Rolling disk method with Quantity One (Bio-Rad laboratories Inc.), which normalizes the band pattern from electrophoresis for identification of each band. We then converted the band patterns into binary data based on the presence or absence of each band for part of our analysis. The DGGE fingerprints were interpreted in terms of band richness (number of predominant DGGE bands/population). The pixel intensity of each band was detected by Quantity One software and is expressed as relative abundance (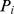) [30]. Shannon Index (*H′*) and Simpson index (*D*), the most widely used diversity indices were then calculated using the richness and relative abundance data following the equations:
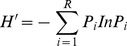
(1)

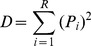
(2)


Where *R*, the richness, is number of different bands each data set contains, 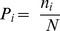 and 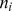 is the abundance of the *ith* band and *N* the total abundance of all bands in the sample.

Band-type data of the DGGE fingerprints was then used to derive the alpha diversity (bands per sample and ecosystem type), beta diversity (total bands per site) [31]. Jaccard's similarity indices [32] between converted and natural TRF sites were determined using the equation:

Jaccard's Similarity Index
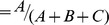


Where,

A  =  Total number of bands present in both converted (C) and natural (N) ecosystem samples (plots) (also β-diversity)

B  =  Number of bands present in C but not in N

C  =  Number of bands present in N but not in C

We determined the influence of site factors as revealed by soil physicochemical properties on the variation of soil microbial communities by applying discriminant analysis using Statistical Package for the Social Sciences (SPSS). This was done to assess the relative importance of each predictor variable (pH, SOC, N, P, K, Na, Ca, Mg, and soil texture). We also used the Mann-Whitney test to examine differences between soil properties in natural and converted ecosystems, and microbial communities in natural and converted ecosystems.

**Figure 2:**
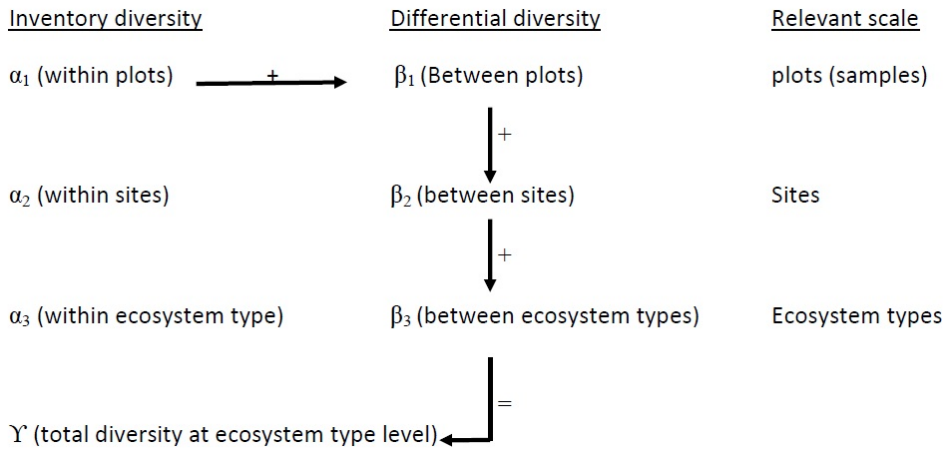
Illustration of hierarchical spatial scales in our additive partitioning model. The α scale is the within-level, and β scale, the between-level components. Because a diversity at a given scale is the sum of the α and β diversity at the next lower scale, the total diversity (γ) can be described by the following formula: α1+β1+β2+β3 [14], [22].

We tested the null hypothesis that diversity is uniform at all spatial scales by additive partitioning of total diversity (γ diversity). To determine contributions of α and β diversity to overall diversity across a range of spatial scales [14], [23], [33], an additive relationship between diversity components (i.e., β  =  γ - α) was derived (Fig 2.). The scale at which diversity is maximized was therefore identified [23], [34], to facilitate planning processes and management strategies to conserve natural levels of diversity accordingly [35]–[38].

We used PARTITION 3.0 software [39] to calculate average diversity at each scale and diversity was measured as band richness. Individual-based randomization procedure in the software was used to test whether the observed partitions of diversity within the ecosystem could have been obtained by a random allocation of lower-level samples nested among higher-level samples [34]. Null values of β_i_ obtained from 1,000 randomizations were used to obtain a *p*-value for the observed β_i_ at each hierarchical scale. Deviations of the observed diversity from the null expectation indicated a nonrandom spatial distribution of fungi or bacteria at a given scale.

## Results

### Soil property variations

Soil pH comparisons using a Mann-Whitney U test of significance, between five plots of natural and five plots of converted TRF ecosystems in each of the four forest sites, found significantly higher (less acidic) pH in three of the four sites at Budongo (*p* = 0.0107), Kaniyo Pabidi (*p* = 0.0112), and Mabira (*p* = 0.0269); and non-significant difference at Maramagambo (*p* = 0.1706). Percentage soil organic carbon (SOC) was significantly higher in natural than converted ecosystems in all four sites i.e. Budongo (*p* = 0.0119), Kaniyo Pabidi (*p* = 0.0212), Mabira (*p* = 0.0122) and Maramagambo (*p* = 0.0119) with combined %SOC in natural sites more than double of that in converted sites; whereas %soil nitrogen was only significantly higher in natural forests at Budongo (*p* = 0.0112) and Kaniyo Pabidi (*p* = 0.0119), and non-significant at Mabira (*p* = 0.6015) and Maramagambo (*p* = 0.0947) (Table 3).

**Table 3:** **Mean (Standard deviation) for diversity indices of Bacterial (B) and Fungal (F) communities and soil properties in natural (N) and converted (C) ecosystems.**

	Budongo		Kaniyo Pabidi		Mabira		Maramagambo	
	Natural	Converted	Natural	Converted	Natural	Converted	Natural	Converted
Shannon (B)	2.49(0.68)	2.73(0.10)	3.25(0.21)	3.12(0.25)	3.06(0.18)	3.19(0.18)	3.04(0.16)	2.93(0.07)
Simpson (B)	0.89(0.10)	0.93(0.01)	0.95(0.13)	0.95(0.02)	0.95(0.01)	0.95(0.01)	0.95(0.01)	0.94(0.01)
Shannon (F)	2.44 (0.30)	2.49 (0.17)	2.83(0.19)	2.77(0.20)	2.57(0.62)	2.64(0.30)	2.10(0.50)	1.93(0.67)
Simpson (F)	0.90(0.29)	0.90(0.03)	0.93(0.01)	0.93(0.02)	0.90(0.06)	0.92(0.03)	0.84(0.08)	0.82(0.12)
pH	5.88(0.11)*	5.08(0.20)*	6.24(0.23)*	5.38(0.27)*	6.46(0.54)*	6.18(0.42)*	6.18(0.45)	5.80(0.24)
OC (%)	6.14(0.67)*	1.59(0.17)*	5.69(1.20)*	3.65(0.54)*	5.87(2.03)*	3.53(1.19)*	9.08(0.79)*	3.98(1.09)*
N (%)	0.43(0.05)*	0.11(0.01)*	0.43(0.08)*	0.25(0.03)*	0.21(0.10)	0.22(0.06)	0.28(0.04)	0.19(0.08)
Ca (Cmoles/kg)	8.00(2.56)*	4.00(0.58)*	13.54(3.36)*	6.74(0.98)*	8.14(1.19)*	6.20(0.76)*	12.12(2.13)*	7.34(1.16)*

*significant differences (p<0.05) between natural and converted ecosystems.

### Ecosystem and site comparisons of microbial community diversity

**Figure 3:**
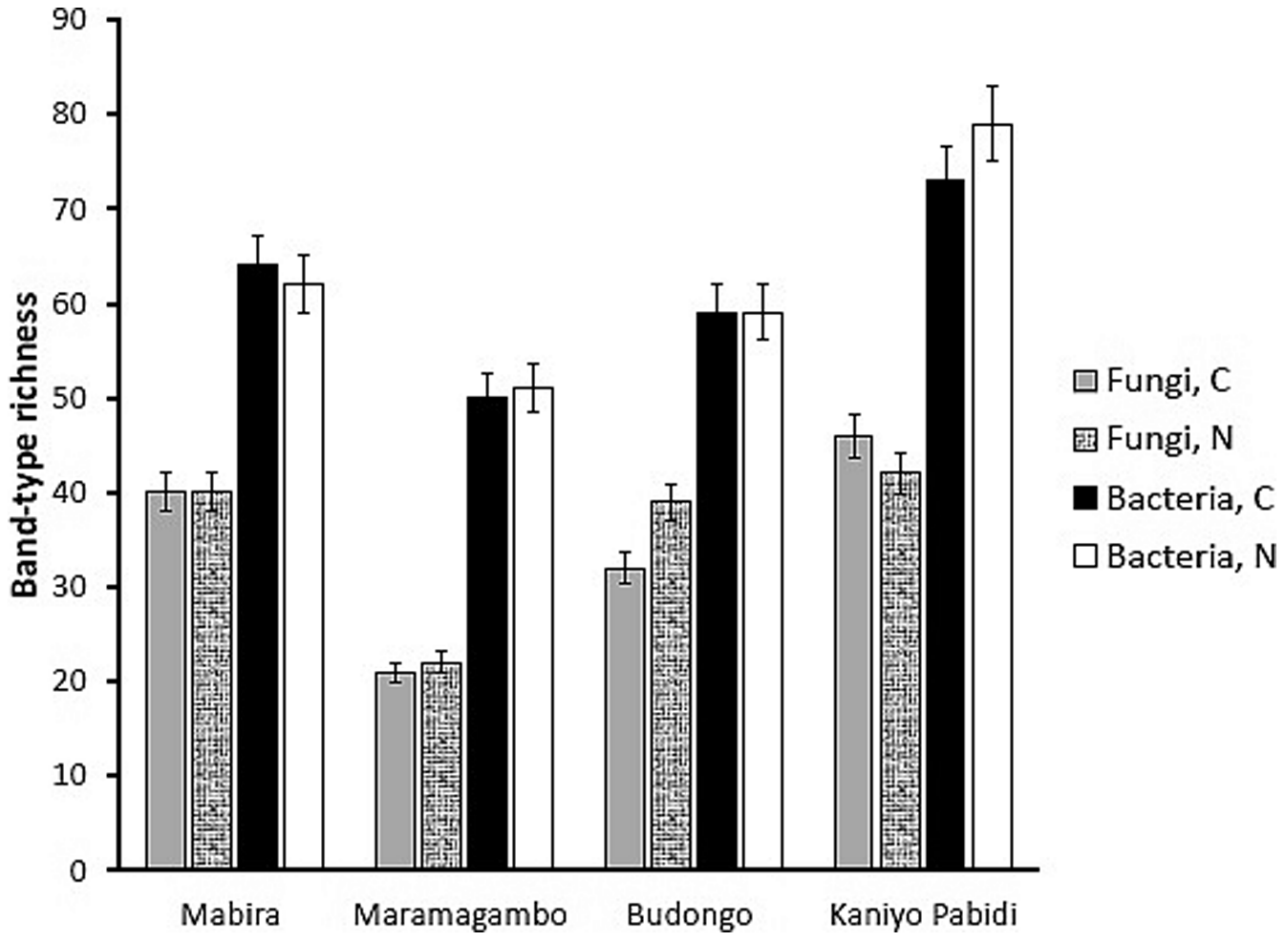
Band richness for fungal and bacterial communities in converted and natural ecosystems. All richness values are total bands present in five samples of each ecosystem treatment (error bars are 5% confidence interval).

Bacterial (B) communities were significantly richer (*p* = 0.0304; Mann-Whitney U) in detectable bands than fungal (F) communities in both converted (C) and natural (N) ecosystems (converted: medians; F = 36, B = 61.5; natural: medians; F = 39.5, B = 60.5). While total band richness (B+F) did not differ between natural and converted forests we observed greater fungal richness in natural than in converted forests (medians: C = 36, N = 39.5; test stat = 18.5) and more bacterial bands in converted than in natural ecosystems (medians: C = 61.5, N = 60.5; test stat = 18.5). Kaniyo Pabidi was the most diverse site overall with the highest number of bacterial and fungal bands, while Maramagambo had the least band richness (Fig 3).

**Figure 4:**
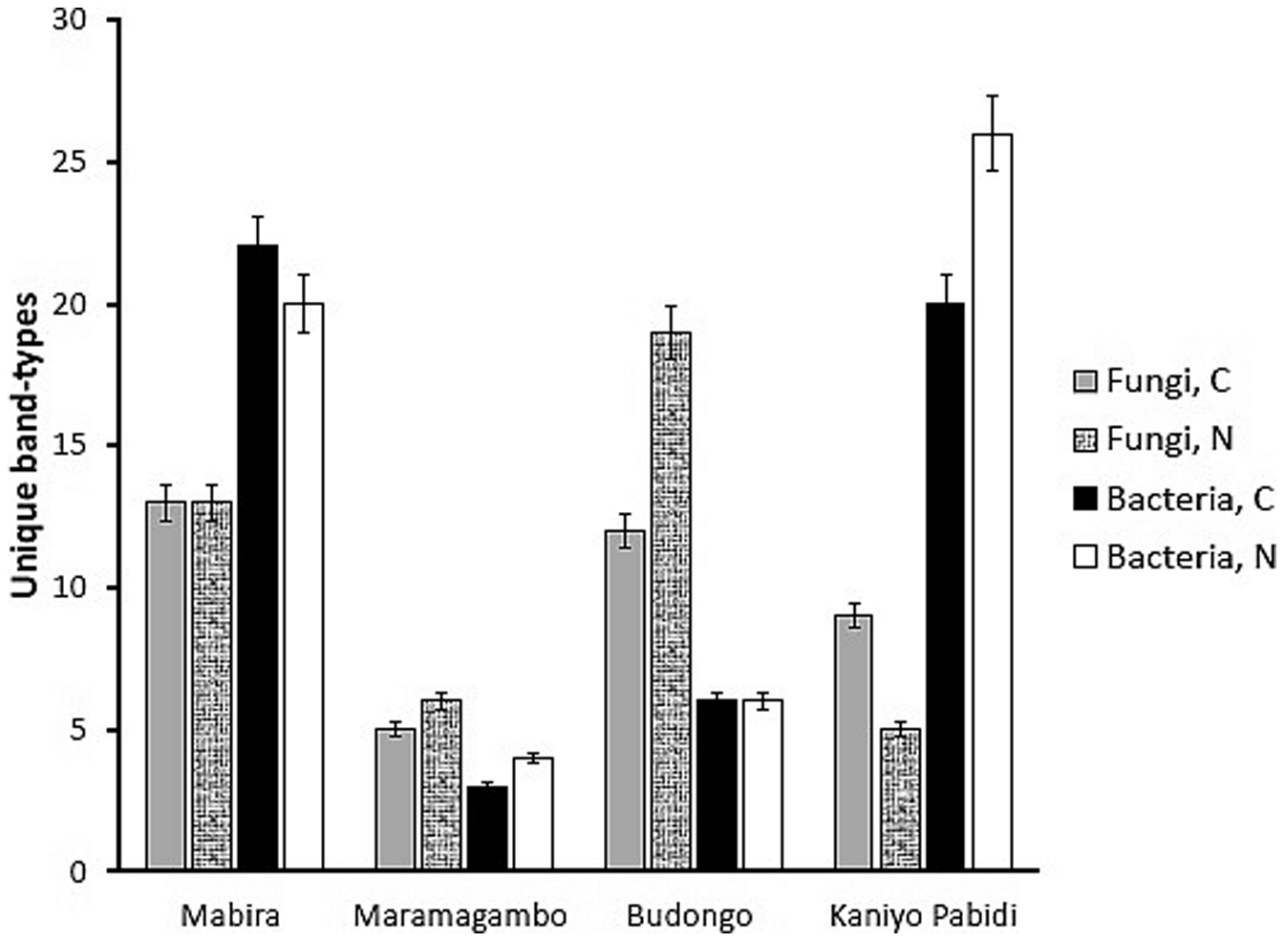
Bacterial and fungal bands unique to converted (C) and natural (N) ecosystems at each site (error bars are 5% confidence interval).

Natural sites harbored more bands unique to one site than converted sites for bacteria at Kaniyo Pabidi and Maramagambo and for fungi at Maramagambo and Budongo. Mabira and Kaniyo Pabidi had higher numbers of unique bacterial bands than at Maramagambo and Budongo. There were also more unique fungal bands at Mabira and Budongo than at Maramagambo and Kaniyo Pabidi (Fig 4).

We also found that Mabira and Maramagambo had the lowest bacterial Jaccard's community similarity indices [32] between natural and converted ecosystems, whereas Budongo and Mabira had the lowest fungal community similarity between natural and converted ecosystems (Table 4). Dissimilarity between natural and converted ecosystems was nonetheless non-significant in all sites for both fungal and bacterial communities. Also, there was generally greater dissimilarity between sites of fungal communities than in bacterial communities suggesting a higher susceptibility to habitat change among fungi than bacteria (Table 4).

**Table 4:** **Jaccard's similarity indices between bacterial and fungal communities in natural (N) and converted (C) sites of Mabira (Mb), Maramagambo (Mg), Budongo (Bd) and Kaniyo Pabidi (Kp).**

			F		U		N		G		I
			*Mb*	*Mg*	*Bd*	*Kp*	*Mb*	*Mg*	*Bd*	*Kp*	
			N	N	N	N	C	C	C	C	F
B	*Mb*	N	1	0.713	0.769	0.732	0.671	0.685	0.696	0.768	
A	*Mg*	N	0.841	1	0.733	0.666	0.666	0.711	0.646	0.666	U
C	*Bd*	N	0.803	0.840	1	0.804	0.808	0.693	0.622	0.827	
T	*Kp*	N	0.731	0.765	0.698	1	0.774	0.670	0.794	0.785	N
E	*Mb*	C	0.667	0.788	0.739	0.741	1	0.663	0.710	0.761	
R	*Mg*	C	0.866	0.885	0.846	0.755	0.809	1	0.684	0.698	G
I	*Bd*	C	0.825	0.857	0.844	0.696	0.731	0.856	1	0.786	
A	*Kp*	C	0.805	0.869	0.716	0.683	0.759	0.776	0.733	1	I
			B	A	C	T	E	R	I	A	

### Ecosystem classification and importance of predictor variables

**Figure 5:**
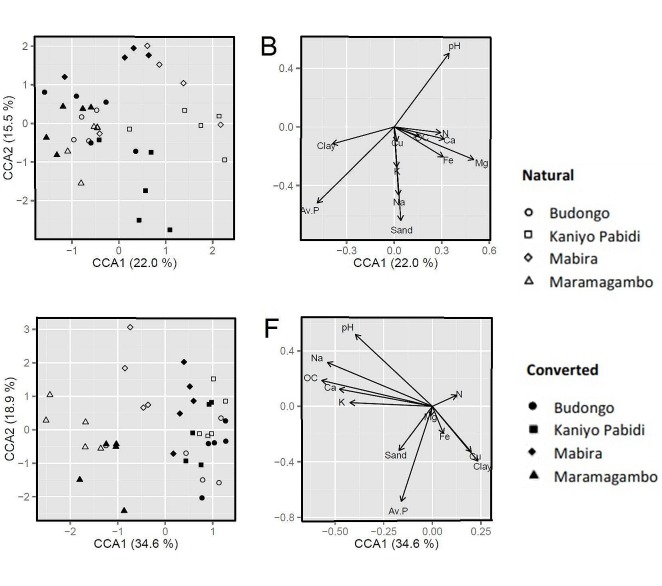
CCA for bacterial (B) and fungal (F) relationships using relative abundance of bands and soil physicochemical properties in natural and converted ecosystems. The symbols (left graphs) represent the similarity between each sample (plot) as defined by their diversity, and the vectors (right graphs) represent the structural matrix for soil properties and their influence on relative abundance of each band. The length of the vectors represents the relative strength of influence of the particular aspect of soil physicochemical property.

The CCA showed that, despite the relatively small amount of difference between sites, soil pH, average phosphorus, and texture (%sand) had strong influence on bacterial diversity in the TRF ecosystem (Fig 5); whereas organic carbon, sodium, pH and average phosphorus were strongly associated with fungal community variation in both natural and converted TRF ecosystems (Fig 5). The CCA also showed that bacterial communities in both Kaniyo Pabidi and Mabira were unique to bacterial communities in the other sites and there was high contrast between bacterial communities of converted and natural ecosystems at Kaniyo Pabidi. Fungal communities at Maramagambo and Mabira were also unique to those in other sites and there was high contrast between fungal communities at Mabira's natural and converted ecosystems. Furthermore, the CCA showed that fungal communities responded more to soil pH levels than bacterial communities (Fig 5), with site-specific patterns showing that bacteria and fungi were grouping according to sites.

A discriminant analysis to predict whether bacterial or fungal communities were from natural or converted ecosystems found that only OC, Ca, N, and pH for bacterial communities; and OC, N, Ca, and pH for fungal communities (all ranked from most important to least important) were found to be significant predictors of soil physicochemical properties. All other variables were poor predictors in this context (Table 5).

**Table 5:** **Structure matrix rank showing absolute size of correlation between discriminant analysis function from most important to least important predictor variable of site factors (soil physiochemical properties) and their influence on the variation of soil microbial communities.**

Bacteria		Fungi	
Predictor Variables	Function 1	Predictor variables	Function 1
OC	0.625*	OC	0.625*
Ca	0.471*	N	0.493*
N	0.421*	Ca	0.473*
pH	0.355*	pH	0.340*
Mg	0.298	Mg	0.281
Cu	0.197	Cu	0.221
Na	0.181	Na	0.183
K	0.169	K	0.160
Av.P	−0.077	Sand	0.064
Sand	0.070	Av.P	−0.060
Simpson	−0.065	Fe	0.024
Fe	0.029	Shannon	0.012
Shannon	−0.023	Simpson	0.011

(* = important predictor variable, with 0.30 used as the threshold).

### Hierarchical scaling

**Figure 6:**
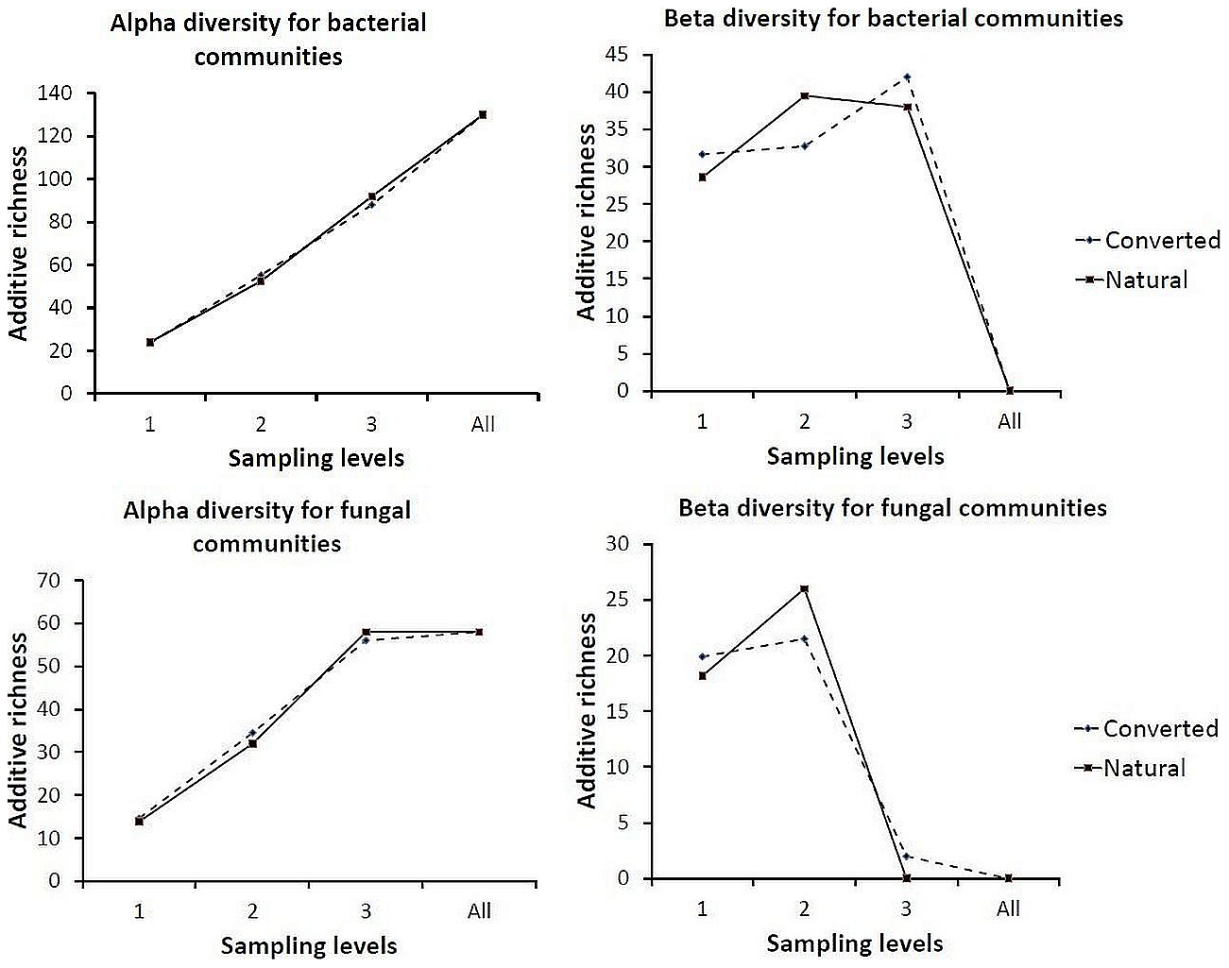
Additive partitioning of bacterial and fungal diversity (expressed as additive richness) across alpha and beta hierarchical spatial scales at three sampling levels (plot, site and ecosystem type) in natural and converted TRF ecosystems.

We found 58 and 56 fungal bands in natural and converted forests respectively, from 17 plots of natural ecosystems and 20 plots of converted forests. There were also 92 and 88 bacterial bands in natural and converted ecosystems respectively found in 20 plots of converted ecosystems and 17 plots of natural ecosystem. All these were within four sites. β-diversity varied more than α-diversity between natural and converted ecosystems for both bacteria and fungi. We found higher bacterial and fungal β-diversity in converted ecosystems than in natural ecosystems at lower hierarchical scales (β_1_); higher β-diversity in natural than converted at between-site scale (β_2_), and higher β-diversity in converted than in natural ecosystems at the between-ecosystem type scale (β_3_) (Fig 6).

**Figure 7:**
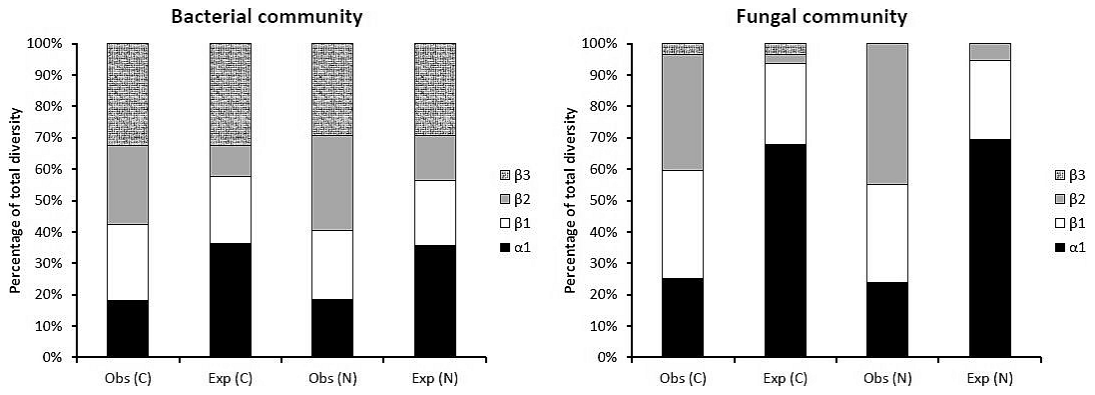
The additive partitioning of total bacterial and fungal community, γ -diversity into α and β components at three nested spatial scales, with each component expressing their relative contributions to γ –diversity; where γ –diversity is equal to α1+β1+β2+β3. The observed (obs) partitions are compared with the expected (exp) values, as predicted by the null model based on 1000 iterations using individual-based randomization.

We also found substantial contribution of observed β-diversity (β_1_, β_2_, and β_3_) to total band richness (γ-diversity), while α-diversity of both bacteria and fungi in converted and natural ecosystems were similar. Spatial partitioning of total diversity also consistently showed that the beta components (β_1_ and β_2_) were always greater than expected by chance, whereas the alpha component (α_1_) was always lower than expected. For both fungal and bacterial communities in natural and converted ecosystems, observed within plot diversity were substantially less than values expected from individual-based randomizations (Fig 7).

## Discussion

### Soil property variations and site differences

Studies in both tropical and temperate zones show that soils in converted or cropped areas normally have reduced soil aggregation, structural stability and organic matter, and an increase in bulk density when compared to forests [40], [41]. Habitat conversion may also alter soil properties such as nutrient levels, and abiotic conditions and may affect associations between organisms. In our study there are some local details that may influence our results.

Both Maramagambo and Kaniyo Pabidi are located within Queen Elizabeth NP and Murchison Falls NP respectively and are protected by Uganda Wildlife Authority (UWA) personnel. They are well protected and there is little evidence of recent encroachment. There is significant wildlife populations including elephants, buffaloes, zebras and the areas are frequented by tourists. Protection by UWA and presence of dangerous animals (such as buffalos and lions) reduce damaging human activity at Kaniyo Pabidi and Maramagambo which should enhance the difference between natural and converted ecosystems. Maramagambo's location, in contrast, means the forest is subjected to substantial natural disturbance from frequent storms and strong erosive runoff even within the natural forest, whereas tourist activity at Kaniyo Pabidi seemed to have little impact on soil properties. Converted areas at Kaniyo Pabidi were also sparsely populated with limited human impacts on the environment. Its sites were old and might have been cultivated for at least 20years.

In our study, Budongo is located next to a high, mainly subsistence population and resultant population activity. But even though encroachment, illegal hunting and logging in natural habitats in Budongo are not uncommon, there seems to be minimal impact of conversion on soil properties in our sample locations; whereas proximity of Mabira's natural forest to densely populated urban areas exposes it to increased human activities, likely reducing its difference with converted sites.

### Microbial community variations

Soil properties determine many aspects of soil microbial community structure [42]–[44]. Carbon availability [45]–[47], nitrogen availability [45], [48], [49] and soil pH [44], [50], [51] can all influence microbial community composition and diversity. In addition, correlation studies have shown that plant species [52]–[54] and soil type [45], [54], [55] are associated with variation in microbial communities. It has also been shown that land use indirectly affects bacterial community structure by modification of soil properties [56] but similarity between converted and natural ecosystem bacterial communities may also suggest a high number of generalists.

Nacke et al., [56] found that bacterial community composition in forests and grassland was largely determined by tree species and soil pH. Jesus et al. [6] also showed that bacterial community structure is influenced by changes linked to soil acidity and nutrient concentration. Other studies also suggest that soil pH is a major factor influencing microbial community composition [50], [57]–[59]. This influence of soil pH has been recognized at different taxonomic levels [45], [60] with most microorganisms thriving within a limited pH range. This is because acids can denature proteins and large pH changes may inhibit growth in microorganisms. Fierer and Jackson, 2006 [44] found, in contrast, that net carbon mineralization rate (an index of C availability) was the best predictor of phylum-level abundances of dominant bacterial groups, and Bisset et al., [42] found that soil microbial communities were consistent with disturbance gradients within different agricultural treatments and relatively undisturbed non-agricultural sites.

Because of widespread forest conversion in Uganda as a result of increasing population pressure, estimated at between 1.1% and 3.15% per year [61]; natural ecosystems and their inhabitant biodiversity are at risk [62]. Loss of diversity increases the likelihood of losing important functional roles and associated ecosystem processes. At landscape scale, spatial and temporal variations of microbial communities in forest soils are influenced by numerous biotic and abiotic factors. These factors may include climate, soil types, and vegetation associations [50], [63], [64]. Owing to this study design, many of these factors were assumed to be similar between natural and converted ecosystems. For instance, the proximity of natural and converted ecosystem sites meant that climate and geology were, we assume, similar in the two treatments. Even though there could still be a number of underlying causes of community differences, two likely influences were assumed to be soil properties [45], [54], [55] and vegetation types [52]–[54]. Clearly both of these sets of factors change when forest is converted for agriculture or range lands.

Despite substantial reductions in SOC, N, Ca and pH in converted sites in this study, differences in microbial communities were small meaning that converted sites still had sufficient SOC, N and Ca to sustain the same microbial populations. The close proximity of the matched pairs could also lead to a source-sink relationship between the natural and converted forests, with the presence of unique taxonomic groups a likely indication of habitat preference (endemism) for some taxonomic groups. It may also be an indicator of relative habitat dissimilarity. The high numbers of unique bacterial bands at Mabira and Kaniyo Pabidi and unique fungal bands at Mabira and Budongo (Fig 4) thus suggests that ecosystem alteration at these sites was sufficient to force a different regime of processes and structures enabling a new set of taxonomic groups to predominate. Mabira had high numbers of both unique bacterial and unique fungal bands that can be attributed to the extent of disturbance at its sites (Mabira is the only peri-urban tropical rainforest site among the four selected sites).

The low numbers of unique bacterial and fungal bands at Maramagambo can be attributed to the high erosion at natural sites that reduced the contrast between the natural and converted sites. For the other sites, bacteria and fungi had different responses to ecosystem alterations. This could indicate separate influences on microbial distribution that exist when alteration is moderate. Similarity indices suggested that bacterial and fungal communities were determined by separate forces leading to distinct responses across the study locations.

### Hierarchical scaling

Many studies have shown that specialist species are more negatively affected by current global changes than generalists [7], [65]. The process of biotic homogenization can involve the replacement of native biota with non-natives or the introduction of generalist species [66]. In this study, the net decrease in β-diversity from natural to converted TRF ecosystem at the between-site scale (β_2_) for both fungi and bacteria was an indication of biotic homogenization [18], [66]. This can result from ecosystem alterations which can in-turn alter ecosystem function and reduce ecosystem resilience to disturbance [65], [67].

We also showed that the β components of diversity (β_1_and β_2_; the average diversity between the plots and sites, respectively) were consistently higher than those expected by chance, whereas the local α_1_ diversity component (α_1_, the average diversity within the plots) was consistently lower than that expected (Fig 7). Such scale-dependent deviations of the observed diversity from the expected can be generally explained by aggregation at a relatively small “local” scale and, spatial differentiation of diversity at a larger “landscape” scale [33], [34], [68], [69].

Relatively lower diversity within converted ecosystems suggests that conversion of natural TRF ecosystems results in reduced diversity for both bacteria and fungi. This is compatible with recent studies that show that conversion of TRF ecosystems threatens microbial diversity [7] and because microorganisms, like all other organisms, have habitat preferences and may be affected by land-use changes [6], [64]. While we cannot be certain that such decline in diversity has led to a decline in any particular ecosystem functions or services, this is a possibility that deserves further evaluation, and we speculate that such loss of diversity will at the very least cause a reduction in functional redundancy and associated resilience.

Higher β-diversity of both bacterial and fungal communities at the between-plot scale (β_1_) in converted ecosystems indicates differentiation (reduced community similarity) in converted ecosystems at this hierarchical scale. Considering the multiple land-uses and cropping systems of converted areas, this was expected. There was also substantial contribution of β-diversity to total diversity (γ). This suggests the importance of nonrandom ecological processes at the between-plot and between-site scale in determining total richness and community composition [14], [34]. Differences between the observed and expected diversity components could be due to ecological processes that lead to a non-random dispersion of individuals. These processes could include intra-specific aggregation, habitat selection, and limited dispersal capacity [33].

## Conclusion

There is international concern about the threat to natural habitats in the Nile river watershed and the consequential loss of important biodiversity. Whereas aspects of microbial biogeography and influence of forest conversion in Uganda's Nile river watershed is largely unknown, this study offers an important first glimpse into indicators of spatial diversity patterns of soil fungal and bacterial communities in the Uganda's Nile river watershed. Our observations of reduced soil microbial diversity, both bacterial and fungal, in converted ecosystems though unsurprising in itself causes us some concern and would justify further work to determine the significance of the diversity lost and the wider implications.

By focusing on diversity patterns across multiple hierarchical spatial scales, we were able to identify the scale at which regional microbial diversity is maximized. We showed that there was substantial contribution of β-diversity to total ecosystem diversity (γ) which includes taxa at the between-plot, site and ecosystem scales and unique taxa, highlighting the necessity to conserve marginal habitats and ecotones. Soil microbial communities in Uganda's Nile river watershed exhibit considerable resilience to forest conversion even though SOC, N, Ca and pH were all significantly altered. This result is surprising given that these physical and chemical properties typically strongly influence microbial diversity. Additionally, the variation among sites was quite large, indicating that soil communities in this region vary considerably on a regional spatial scale. Our results do not explain this variation. Most studies suggest that biogeographic barriers play little role in the geographic structure of soil communities. Rather than a consistent general pattern of microbial community change following forest conversion we find that responses are largely site-specific and widely variable.

## Acknowledgments

We confirm that our field work did not involve any endangered or protected species and that we were granted access to protected areas by Uganda Wildlife Authority (UWA) and National Forestry Authority (NFA). Our sample site coordinates included: (1) Mabira; Lat, N00°24.495'; Long, E033°02.464' (Required permission obtained from NFA); (2) Maramagambo; Lat, S00°04.421'; Long, E030°02.305' (Required permission obtained from UWA); (3) Budongo; Lat, N01°41.852'; Long, E031°29.363' (Required permission obtained from UWA/NFA); (4) Kaniyo Pabidi; Lat, N01°55.128'; Long, E031°43.116' (Required permission obtained from UWA).

We would like to thank the Institute of Tropical Forest Conservation (ITFC) of Mbarara University of Science and Technology for supporting and co-advising this research. We also thank the Genetics Lab at Faculty of Science and the soil department lab at Faculty of Agriculture both at Makerere University, Kampala, for their lab facilitation during extractions and analyses. And finally we acknowledge the ideas and insights of Masatoshi Katabuchi, Dossa Gbadamassi, and our field and lab technicians and assistants Solomon Echel, Francis Alele Ozirit and Boniface Balikuddembe.

## Supporting Information

Data S1 Excel spreadsheet from Quantity One analysis of PCR-DGGE profiles of fungal and bacterial communities in natural and converted TRF ecosystems.
(XLSX)
